# Single‐use IgE‐selective immunoadsorber column for the treatment of severe atopic dermatitis

**DOI:** 10.1002/jca.21759

**Published:** 2019-11-22

**Authors:** Damian Meyersburg, Martin Laimer, Andrea Kugler, Eva Mühlthaler, Nadja Lindlbauer, Wolfgang Hitzl, Eva Rohde, Johann W. Bauer, Christoph Grabmer

**Affiliations:** ^1^ Department of Dermatology University Hospital Salzburg of the Paracelsus Medical University Salzburg Salzburg Austria; ^2^ Department of Transfusion Medicine Paracelsus Medical University Hospital Salzburg Salzburg Austria; ^3^ Spinal Cord Injury & Tissue Regeneration Center Salzburg (SCI‐TReCS) Salzburg Austria; ^4^ Research Office (biostatistics) Paracelsus Medical University Salzburg Salzburg Austria; ^5^ Department of Ophthalmology and Optometry Paracelsus Medical University Salzburg Salzburg Austria; ^6^ Research Program Experimental Ophthalmology and Glaucoma Research Paracelsus Medical University Salzburg Austria

**Keywords:** atopic dermatitis, IgEnio, immunoadsorption

## Abstract

**Background:**

Atopic dermatitis (AD) is a chronic and relapsing inflammatory skin disease with unmet therapeutic need in a critical cohort of recalcitrant cases. Immunoadsorption (IA) aims at an immunomodulatory depletion of pathogenic serum mediators and has recently revealed promising clinical results for the treatment of AD.

**Objective:**

To determine efficacy, sustainability, safety, and clinical impact of IgE selective IA in AD using a single‐use IgE immunoadsorber column.

**Methods:**

This open‐label pilot study comprised five patients (mean SCORAD 67.9 ± 11.4, range 52.2‐81.9; mean serum IgE level 5904 ± 5945 U/mL, range 1000‐15 600 IU/mL) who underwent IgE‐selective IA. Three patients continued prior therapy with systemic immunosuppressive drugs during IA as an add‐on therapeutic approach. All patients received three courses of IA. The first course consisted of three consecutive daily treatments followed by two sequences with two consecutive applications. All courses were performed on a monthly regimen.

**Results:**

IA proved efficacy in selectively depleting serum IgE levels in all participants (mean reduction by cycle of 81% ± 12%, range 64%‐93%). It further led to a clinically relevant and sustained improvement of AD with a maximum decline in SCORAD and EASI scores by up to 35% and 52%, respectively, compared to baseline. Scores persisted below baseline for at least 12 weeks beyond the last IA. The intervention was also well tolerated with no severe adverse events during a total of 35 procedures.

**Conclusion:**

Data of this preliminary trial indicates clinical efficacy, feasibility, safety as well as tolerability of IgE‐selective IA in AD.

## 1 INTRODUCTION

Atopic dermatitis (AD) is a chronic and relapsing inflammatory skin disease characterized by intense itching and eczematous skin lesions that, in developed countries, affects 20%‐30% of children and about 10% of adults.^1^ In severe or refractory disease, systemic immunosuppressive drugs, that is, cyclosporine, methotrexate, azathioprine, or mycophenolate mofetil, have been used with inconsistent therapeutic responsiveness and/or tolerability.^1^ The therapeutic armamentarium for advanced AD has been enriched by the monoclonal (IL‐4 and IL‐13) antibody dupilumab, which was reported to achieve a 75% eczema area and severity index (EASI) improvement from baseline in 44% to 51% of patients by targeting key inflammatory pathways involving interleukin 4 and 13.^2^

Despite these advances and further promising experimental agents including JAK inhibitors and anti‐IL‐12, −13, −31R, or − 22 as well as histamine 4 receptor antibodies ahead, there is currently a highly unmet therapeutic need for a critical cohort of recalcitrant AD cases.^3^

Patients with AD commonly have elevated total IgE levels (extrinsic AD). Their distinct role in the complex pathogenesis of AD, however, remains unknown.^4^ Somewhat consistently, data on the clinical efficacy of omalizumab, a recombinant humanized monoclonal antibody targeting the high‐affinity Fc receptor of IgE, are conflicting in AD, even when focusing on the subgroup of patients with high IgE levels. Thus, omalizumab failed to improve AD symptoms or revealed non‐superiority to placebo in several clinical trials.^5^, ^6^, ^7^, ^8^, ^9^, ^10^, ^11^

Immunoadsorption (IA), aiming at an immunomodulatory depletion of pathogenic serum mediators, was previously reported to cause disease amelioration paralleled by normalization of cutaneous inflammatory parameters (ie, density of CD3^+^, CD4^+^, CD1a^+^ cellular infiltrates) in AD patients refractory to multiple conventional treatment strategies including cyclosporine A. While different protocols have been used applying IA either with pan‐Ig (four studies, 53 patients)^12^, ^13^, ^14^, ^15^ or IgE‐selective adsorbers (three studies, 38 patients)^13^, ^16^, ^17^ as well as in combination with omalizumab (one study, 10 patients), all these regimes were reported to induce a significant improvement of disease severity in the majority of patients (and partly irrespective of IgE load) for at least 3‐6 months beyond therapy.

Herein we present a series of patients with severe AD and significant elevated serum IgE who underwent IA using an IgE‐selective single‐use column. This approach proved clinical efficacy, feasibility, and excellent tolerability.

## 2 MATERIALS AND METHODS

### 2.1 Study design

Inclusion criteria of this prospective single‐center study comprised^1^ severe AD, that is, severity scoring of atopic dermatitis (SCORAD) ≥ 50,^2^ requiring systemic treatment, with previously poor response to (or contraindication for) at least one systemic immunosuppressive therapy (eg, steroids, cyclosporine A, methotrexate, azathioprine, and mycofenolate mofetil),^3^ total serum IgE level > 750 IU/mL,^4^ age of ≥18 years, and^5^ vascular access via either peripheral or central venous catheters. Prior local and systemic immunosuppressive therapy, which had been associated with a limited and clinically unsatisfactory response despite long‐term use (see Table 1), was allowed to be continued during the testing of IA as an add‐on therapeutic approach in recalcitrant cases. Exclusion criteria were^1^ known hypersensitivity or allergy to contents and materials used in the adsorber columns,^2^ contraindication or intolerability to anticoagulation (eg, multiple allergies to various anticoagulants),^3^ bleeding disorders including hypo‐ and hypercoagulability,^4^ severe cardiovascular disease (eg, cardiac failure, New York Heart Association [NYHA] stage ≥III),^5^ systemic infection (including hepatitis B/C, tuberculosis) requiring active treatment,^6^ IgG serum levels <250 mg/dL,^7^ severe immunodeficiency (eg, AIDS),^8^ treatment with angiotensine‐converting enzyme inhibitor that could not be suspended for ≥72 hours prior to IA,^9^ hypocalcaemia, and^10^ pregnancy.

**Table 1:** Patient characteristics

Patient	Gender	Age	Disease duration (years)	SCORAD at enrollment	Serum IgE level at enrollment^a^	Previous therapies and outcome^b^	Concomitant therapy and outcome^b^
1	Male	45.6	32	52,2	6320	CyA (ITR)	MTX 10 mg/week since 2 years (ITR, but stable disease)
2	Male	36.4	36	66,8	15 600	MTX, CyA, MPA, AZA (each ITR)	None
3	Female	38.8	38	81,9	5490	CyA (USE); MPM, RTX, IFX (each ITR)	AZA 100 mg/day since 5 years (ITR, but stable disease)
4	Male	45.6	45	75,5	1110	CyA (USE), MTX (ITR)	MP 4 mg/day (ITR, but stable disease) since 15 years (up to 8 mg/d for three consecutive days in disease flare up)
5	Male	56.5	28	63,2	1000	(contraindication to CyA due to hypertension)	None

Abbreviations: AZA, azathioprine; CyA, cyclosporine A; MP, methylprednisolone; MPA, mycophenolate mofetil; MTX, methotrexate; RTX, rituximab; IFX, infliximab.

a Unit in IU/mL.

b ITR, insufficient therapeutic response; USE, unfavorable side effect.

Written informed consent was obtained from all patients before participation in the study following the Declaration of Helsinki and after approval by the local ethics committee.

Referring to efficacy data of IA and dupilumab as a Reference 2, the primary endpoint of the trial was defined as a ≥ 50% reduction of disease activity from baseline measured by SCORAD and EASI at any measure time‐point after the first IA course during the 29‐weeks study period (9‐week interventional and subsequent 20‐week observational phase). Sustainability as a secondary endpoint was defined as a SCORAD at week 29 that shows an increase of ≤20% compared to the lowest SCORAD value obtained during the entire study period. These definitions also reflect the fact that the impact of the IA schedule on IgE courses and their correlatability with the clinical outcome in the chronically as well as severely affected patient cohort recalcitrant to standard therapy modalities was unknown and not predictable prior to study.

### 2.2 Apheresis and treatment schedule

All patients received three courses of IA using the IgEnio adsorber column (IgEnio, Fresenius Medical Care, Bad Homburg, Germany) which is based on a recombinant, IgE‐specific antibody fragment used for the specific extracorporeal depletion of IgE.^18^ The first course consisted of three consecutive daily treatments followed by two courses with two consecutive treatments. All courses were performed on a monthly regimen (9‐weeks intervention phase) This schedule was defined in an attempt to evaluate the therapeutic impact of IA as well as means to reduce the patients' treatment burden by reducing the duration of an IA‐course compared to previous studies with reusable IA‐columns over five consecutive days.^12^, ^14^, ^16^, ^17^ In all study participants IA procedures were performed via peripheral venous accesses. Patients' plasma was separated from blood cells by centrifugation with an apheresis device (Spectra Optia, Terumo BCT, Lakewood, Colorado) processing the 2‐fold calculated plasma volume during each treatment. The maximum plasma flow rate was 30 mL/min, requiring an average time of 3 to 4 hours to treat the plasma volume of each patient. Anticoagulation during treatment was maintained by continuous citrate dosage at a volume to citrate ratio of 1:18 (ACD‐A, anticoagulant citrate dextrose solution A; Terumo BCT, Lakewood, Colorado), and sodium heparin (Heparin Immuno, Immuno AG, Vienna, Austria) with 70‐80 international units (IU)/kg body weight. Separated plasma was conducted by a plasma pump from the centrifugal separation chamber directly to the IgE‐adsorber. The processed plasma was thereafter passed through a particle filter as secondary safety barrier against accidental particle infusion and reunified with the blood cells in the bubble catcher of the cell separator's return line. From there, it was returned to the patient by a second peripheral venous access at the opposite arm.

### 2.3 Clinical and laboratory examinations

The SCORAD and EASI scores were independently obtained by two experienced dermatologists at baseline and weeks 3, 5, 13, 17, 21, and 29. Additionally, the use of concomitant topical and systemic therapy, adverse events, and Dermatology Life Quality Index (DLQI) were recorded.^4^ At every treatment visit, blood was collected immediately before and after apheresis. Total levels of serum IgE, IgG, IgM, and IgA were analyzed using the UniCAP (Thermo Fisher, Waltham, Massachusetts) and the BN2 Nephelometer system (Siemens, Vienna, Austria). Histological (hyperkeratosis, acanthosis, spongiosis, dermal inflammatory infiltrate) and immunohistological (total skin‐bound IgE, CD3^+^−, CD4^+^−, CD1a^+^− cells) parameters were acquired as part of the study protocol at baseline and week 13 (ie, 4 weeks after the last IA). Baseline specimens were taken from the clinically most affected skin area except for cosmetically or surgically sensitive areas such as facial, genital, and intertriginous sites or décolleté. The same location was re‐biopsied for follow‐up at week 13.

### 2.4 Statistical analyses

Data consistency was checked and data were screened for outliers and normality by using Kolmogorov‐Smirnov tests. Cross tabulation tables with Fisher's Exact test or Pearson's test were used to analyze cross tabulations. Generalized estimation equation models based on Tweedie distributions and the logarithm as link function were used to analyze data. All reported tests were two‐sided, and *P*‐values <.05 were considered as statistically significant. All statistical analyses in this report were performed by use of NCSS (NCSS 10, NCSS, LLC. Kaysville, Utah), STATISTICA 13 (Hill, T. & Lewicki, P. Statistics: Methods and Applications. StatSoft, Tulsa, Oklahoma) and PASW 21 (IBM SPSS Statistics for Windows, Version 21.0., Armonk, New York).

## 3 RESULTS

### 3.1 Patients

Patients' characteristics are summarized in Table 1. Five patients (four males and one female, mean age 44.8 ± 7.8 years, range 36.4‐56.5) with severe AD (mean SCORAD 67.9 ± 11.4, range 52.2‐81.9) and highly elevated serum IgE levels (mean 5904 ± 5945 U/mL, range 1000‐15 600 IU/mL) were enrolled consecutively over a period of in total 6 months. The number of probands was limited due to study budget restrictions. Participants had been severely and chronically ill and had experienced either insufficient disease control or unfavorable side effects along previous immunomodulatory and/or suppressive therapy. Tolerated premedications with limited, clinically unsatisfactory therapeutic response despite long‐term use were allowed to be continued at time of enrollment and during the testing of IA as an add‐on therapeutic approach in these recalcitrant cases. Systemic intake was reported by three of five patients without change in frequency and/or dosage in (ie, systemic antihistaminic [levocetirizine 10 mg/day: n = 1; hydroxyzine 25 mg/day: n = 2; desloratadine 5 mg/day: n = 1] and immunosuppressive therapy [methylprednisolone 4 mg/day: n = 1; azathioprine 100 mg/day: n = 1; methotrexate 10 mg/week: n = 1]), except for patient 4 who changed his daily intake of methylprednisolone from 4 to 8 mg at study week 17 (ie, 8 weeks after the last IA) and back to 6 mg at week 20. Although previously prescribed topical therapies were allowed to be dose‐modified according to disease activity during the intervention and observational phase, no relevant changes in their use were recorded during the study.

### 3.2 Clinical course

#### 3.2.1 Outcome

All five enrolled patients completed the intervention phase. One study participant (P5) discontinued the study during observation at week 21 due to treatment unresponsiveness and steadily high SCORAD and EASI scores. The last value obtained in P5 was not carried on forward, thus statistical analyses at week 29 (follow‐up) included only data of the four remaining patients. Notably, three of five patients continued their prior immunosuppressive therapy during the testing of IA (Table 1). As this medication had been associated with a limited and clinically unsatisfactory response despite long‐term use, IA was intended to serve as an add‐on therapeutic approach in this setting.

#### 3.2.2 Efficacy

**Figure 1:**
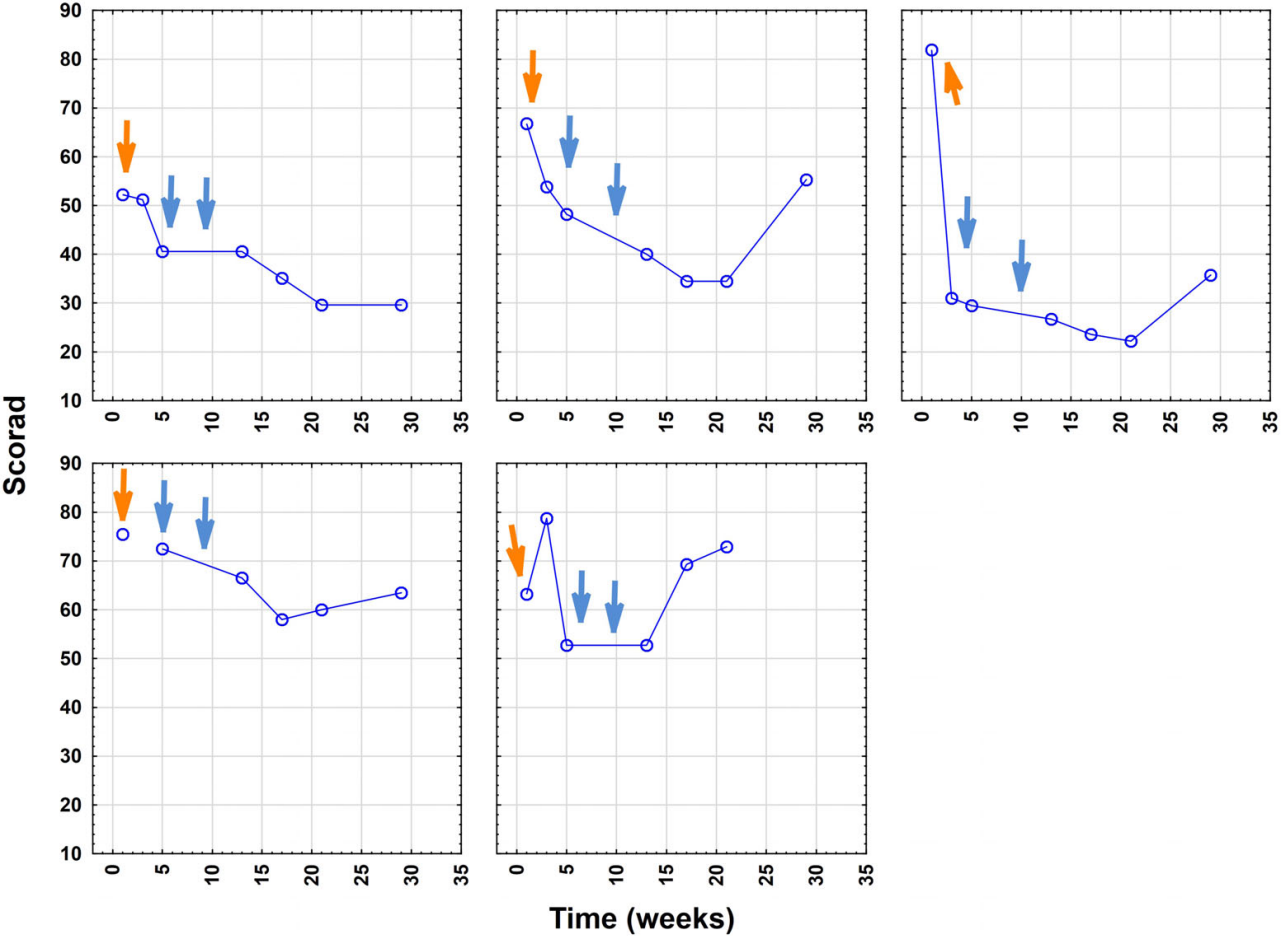
Course of SCORAD during immunoadsorption (IA) in the five study participants (orange arrows indicate first IA cycle with three consecutive treatments on day 1 to 3, blue arrows indicate IA cycles 2 and 3 with two consecutive treatments on day 1 and 2). SCORAD, severity scoring of atopic dermatitis

The primary endpoint, defined as a ≥ 50% reduction in SCORAD from baseline at any measure time‐point after the first IA course during the 29‐week study period, was achieved in two of five patients (ie, 40% of probands), with maximal individual SCORAD reductions ranging from 44% to 84% (Figure 1). The three patients with most elevated IgE levels (mean 9137 ± 5612 IU/mL) showed better responses and a mean decline in SCORAD by 57% to 28.8 ± 6.2 (*P* < .001) as assessed at week 21.

**Figure 2:**
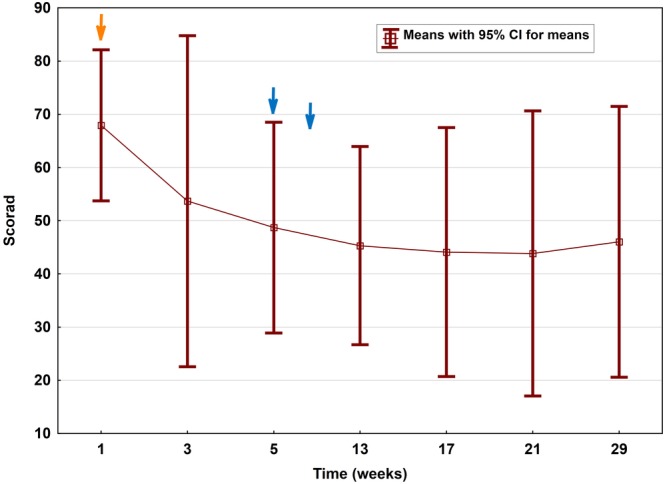
Course of mean SCORAD (P1‐5) along IA treatment (orange arrow indicates first IA cycle on day 1 to 3, blue arrows indicate IA cycles 2 and 3 with two consecutive treatments at week 5 and 9). SCORAD, severity scoring of atopic dermatitis

**Figure 3:**
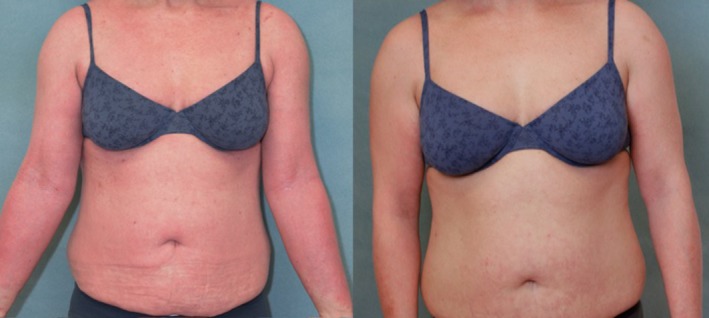
(A, B), Study patient before initiation (week 1; left) and 12 weeks after treatment (right) with 3 cycles of immunoadsorption (ie, seven treatments), depicting a significant clinical improvement with declining redness, swelling, oozing areas (eg, cubital fossa) and scratch marks

**Figure 4:**
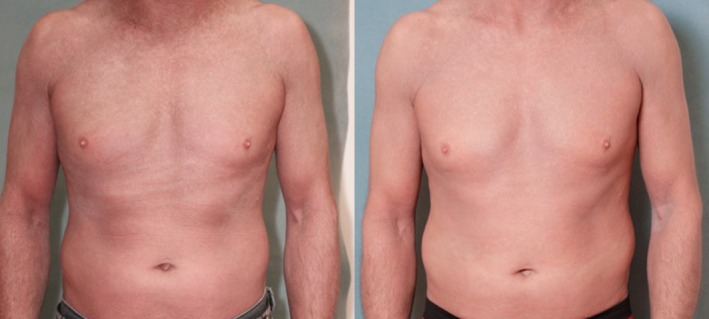
(A, B), Study patient before initiation (week 1; left) and 12 weeks after treatment (right) with 3 cycles of immunoadsorption (ie, seven treatments), showing an amelioration of erythema and swelling

Clinically relevant improvement of mean SCORAD (baseline 67.9 ± 11.4) within the study cohort was first observed at week 5, that is, after the first cycle, revealing a reduction by mean 28% to 48.7 ± 15.8 (*P* = .017). Thereafter, the decrease in mean SCORAD was 33% (to 45.3 ± 15.0; *P* < .05) at week 13; 35% at week 17 (44.1 ± 18.9; *P* < .05) and week 21 (43.8 ± 21.6; *P* < .05); as well as 32% for the remaining four patients at week 29 (46.0 ± 16.0; *P* < .05) (Figure 2). When referring to those three probands with most elevated IgE levels, a significantly better outcome (mean reduction by 32%; mean decline from 67.0 ± 14.9 to 45.3 ± 12.5; *P* = .025) at an earlier stage already at week 3 was noted (followed by minus 41% at week 5 to a mean score of 39.4 ± 9.4 [*P* < .005] and minus 57% at week 21 to a mean score of 28.8 ± 6.2 [*P* < .001]) (Figures 3 and 4).

In parallel with SCORAD, mean EASI decreased significantly by up to 52% at follow‐up week 13 and sustained reduced by 46% (*P* = .009) as assessed at follow‐up week 21. EASI50 (ie, a 50% reduction in EASI scores) was achieved in three of five patients (ie, 60% of probands) during the study period and mean pruritus scores (obtained from patient‐oriented SCORAD grading) decreased by up to 65% (week 17), respectively. Likewise, improvement in DLQI was observed in all five patients (mean 15.6 ± 8.4 at baseline to 6.4 ± 6.4 at week 13 and 12.4 ± 9.3 at week 29).

#### 3.2.3 Sustainability

A rebound, defined at week 29 as an increase of ≥20% from the lowest SCORAD value measured during the study period, was observed in three patients (patients 2, 3, and 5). Two patients (40%) achieved the secondary endpoint. Clinical rebounding occurred earlier in those two patients with the initially the lowest serum IgE burden and was observed in P5 at week 17 and in P4 at week 21 (Figure 1).

#### 3.2.4 Safety

IA was safe and well‐tolerated with no adverse events occurring in any study participant.

### 3.3 Serum immunoglobulin levels and histology

#### 3.3.1 Efficacy

**Figure 5:**
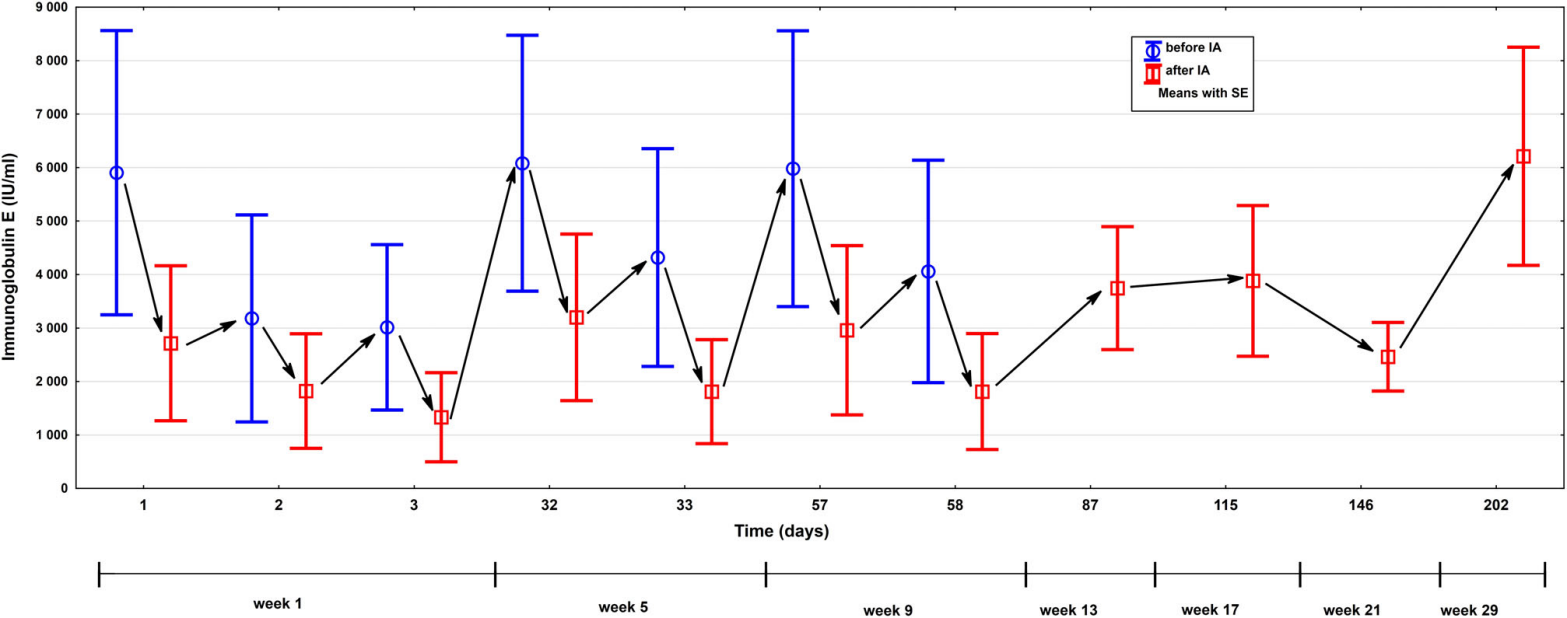
Course of mean serum IgE levels before (blue) and after (red) each treatment (P1‐5) over the course of IA therapy and follow‐up period

Serum IgE levels at baseline ranged from 1000 to 15 600 IU/mL (mean 5904 ± 5945 IU/mL). Each immunoadsorption course reduced total IgE levels by mean 81% ± 12% (range from 64%‐93%). Although IgE levels started to recover ~17 hours after each IA, repetitive apheresis treatments led to a continuous decrease of serum IgE levels in a sawtooth‐like manner (Figure 5). In those patients with initially excessively elevated IgE (n = 3, P1‐3), decrease in IgE levels was higher along the first course comprising three treatments compared to the following courses involving only two treatments (mean IgE‐depletion rates 80% for cycle 1 vs 71% and 72% for cycles 2 and 3). This is supposed to reflect the continuous decline of substrate (IgE serum burden) during therapy as substrate binding characteristics of adsorbers follow the law of mass action (unpublished observation, Fresenius Medical Care Germany). According to the latter, high IgE levels at the start of the treatment (as detected in patients 1‐3) result in high (absolute) amounts of IgE bound inside of the adsorber, whereas the percentual IgE reduction rate in blood would be lower in light of abundant serum IgE. In line with this assumption, slightly better IgE‐depletion‐rates in percentage were observed in study patients with initially lower IgE levels (P4, P5; mean IgE‐depletion rates 95%, 91%, and 91% for cycle 1, 2, and 3).

#### 3.3.2 Sustainability

In the three patients with excessively elevated serum IgE (mean 9137 ± 5612 IU/mL at week 1) its levels stayed below baseline until the last follow‐up visit at week 29 (mean 6213 ± 5612 IU/mL). In contrast, levels returned to initial concentrations already in week 13 or 17 in those two patients with lower serum IgE burden (mean 1055 IU/mL ± 77.8 to 1190 IU/mL ± 161), which was associated with an earlier clinical rebounding as mentioned above. This observation is suggested to reflect a faster recovery of (already initially) lower IgE levels to the baseline value and beyond.

Reductions in total serum levels of IgG, IgA, and IgM (average drop of IgG, IgM, IgA by 9%, 13%, 12%, respectively) occurred within normal lab ranges, were clinically insignificant and only transient (up to 24 hours), followed by a rapid recovery to baseline values.

**Figure 6:**
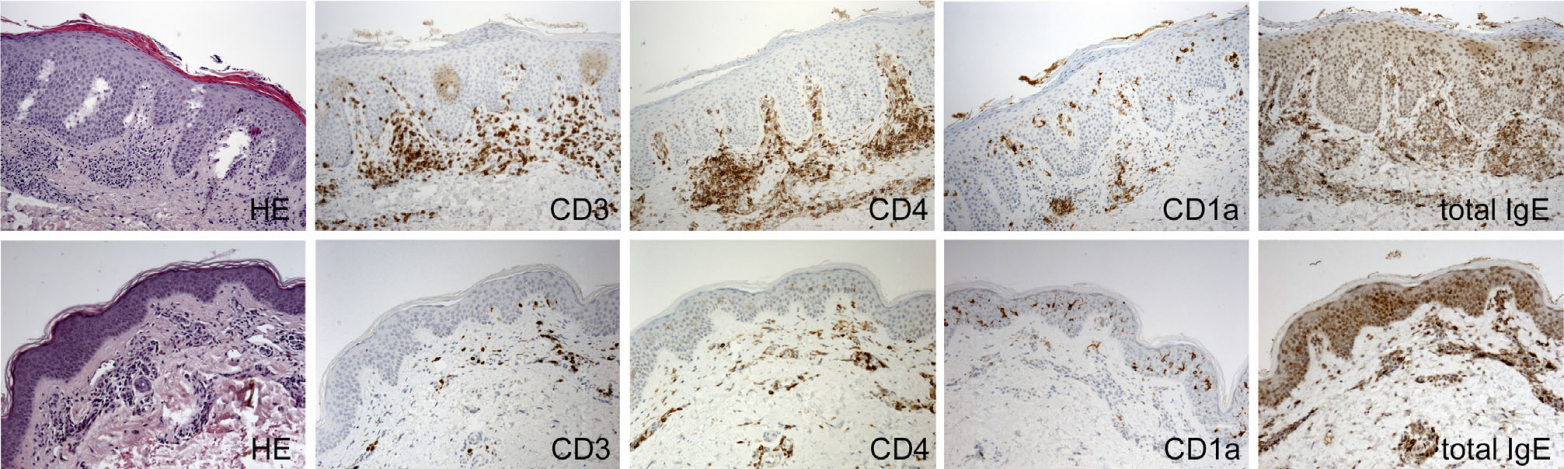
Histological and immunohistological features of skin biopsies taken at baseline and week 13 (ie, 4 weeks after the last IA) indicate a decline in acanthosis, hyperkeratosis, spongiosis and inflammatory cell burden (total skin‐bound IgE, CD3 + −, CD4 + −, CD1a + − cells) in patients responsive to IA. Parameters were not quantified

In those patients clinically responsive to IA therapy, comparative assessment of histological specimens suggested a decrease of hyperkeratosis, acanthosis, spongiosis, and density of (CD3+, CD4+, and CD1a+) dermal inflammatory infiltrate and total skin‐bound IgE from baseline to week 13 (Figure 6; data not quantified).

## 4 DISCUSSION

Our study shows that IgE‐selective IA reduced serum IgE levels by mean 81% per cycle. Although serum IgE levels started to raise again between each IA cycle and in the 20‐week follow‐up period, this intervention was accompanied by a clinically relevant amelioration of AD severity scores (peek reduction in SCORAD and EASI by 35% and 52% form baseline, respectively). For comparison, dupilumab was reported to induce a 75% reduction of EASI in about 50% of AD patients,^2^ Scores persisted below baseline for at least 12 weeks beyond the last IA, an observation that may provide guidance for the determination of an effective maintenance schedule.

Our results further indicate that IA treatment tends to be clinically more efficient in patients with excessively elevated IgE. This subcohort showed more powerful and earlier SCORAD/EASI responses as well as IgE levels that stayed below baseline during the whole study period. Notably, data from previous studies indicate that elevated total serum IgE levels in AD patients correlate with increased prevalence of IgE autoreactivity.^19^ Research also suggests a positive correlation between autoreactivity and AD severity.^19^ In addition, IA selectively depletes only IgE while no simultaneous clearance of other inflammatory serum mediators occurs along the intervention. For the majority of our patients, this would argue for a pathogenic role of IgE that might mediate the induction of downstream pro‐inflammatory pathways finally affecting AD phenotype and severity. However, considering the small number of probands in our study as well as the fact that (the proportion of) IgE‐autoreactivity was not assessed, the pathogenic relevance of our observation, that may independently and individually influenced by for example, various environmental triggers, remains undetermined and elusive.

During the 29‐week study period, IA in our patients showed no significant clinical impact on chronic (post‐acute inflammatory) eczematous skin symptoms and long‐term sequelae such as lichenification or skin atrophy after prolonged application of potent topical corticosteroids. Likewise, dryness of non‐affected skin remained unchanged. All these objective morphological parameters, however, have a significant impact on SCORAD and EASI scoring tools. Our patients had a long history of disease with severe lichenification, erythema and/or dryness. These symptoms may interfere with the assessment of IA efficacy on AD (activity) when using clinical scores within a rather short time interval. In this context it is noteworthy, however, that microstructural analyses, besides a decline in hyperkeratosis, spongiosis and inflammatory cell burden, confirmed a decrease of acanthosis between week 0 and 13 (Figure 6).

A number of studies and case series have already shown efficacy of pan‐Ig and IgE‐selective IA in AD with comparable efficacy.^12^, ^13^, ^14^, ^16^, ^17^, ^20^ Such protocols applied reusable adsorbers that are designed for 10‐20 treatments. To meet functional needs due to limited durability and storability of these adsorbers, study plans applying reusable devices are commonly adjusted to include 4 to 5 days of treatment. Major shortcomings of such regimens include a longer inpatient stay during prolonged courses of apheresis with a higher therapy burden. Against this background, specific single‐use adsorber offer significant advantages in terms of efficacy. First, data indicate that treatment courses may be reduced to 1‐3 interventions as, based on its fast‐acting effectivity, no additional clinical benefit was seen from more treatments in this study. Avoiding problems of storage, stability, shelf life, and risk of bacterial contamination along the use of reusable adsorbers once they have been used for the first time, treatment with single‐use adsorber can be performed flexibly on demand according to patient's disease activity and availability. Another advantage of single‐use adsorber in this study is its combinability with the Spectra Optia apheresis device. The latter is commonly used for the collection of peripheral blood stem cells and thus readily available in most university hospitals in western countries. Moreover, as no additional device for regeneration of the single‐use column is necessary, it can be easily and cost‐effectively combined with different apheresis systems.

IA treatment was well tolerated with no adverse events (AE) occurring in our five patients. This favorable outcome—also with regard to IA‐imminent infectious events^13^, ^21^—may be attributed to the IgE selectivity of the tested column. In addition, based on the vascular status of our probands, IA in this study could be performed using just peripheral instead of central venous catheters, the latter which harbor an increased risk for *Staphylococcus aureus* septicemia^12^, ^16^ or air embolism.^13^ Other common AEs considered to be related to (nonselective, that is, pan‐Ig IA) IA itself in the background of AD (eg, herpes labialis or herpes keratitis caused by HSV‐1; bacterial conjunctivitis caused by *Staphylococcus aureus*; and bacterial sinusitis) were not observed in our study cohort.^13^

One major limitation of this pilot study is the absence of a placebo control. As placebo and nocebo effects were shown to have a considerable impact on disease severity and distinct symptoms of AD our results must be interpreted with caution.^22^, ^23^, ^24^ Likewise, seasonal variations of AD severity could have had an impact on SCORAD as an outcome measure. However, all probands reported a history of chronic and stable severe disease whose course had been recalcitrant to standard therapy modalities and unaffected by determinable exogenous disease modifiers. Three patients continued prior therapy with systemic immunosuppressive drugs during the testing of IA, although these medications had provoked only a limited and clinically unsatisfactory response despite long‐term use before enrollment (Table 1). In addition, patient 4 increased his daily intake of methylprednisolone from 4 to 8 mg during the follow‐up period at study week 17 (ie, 8 weeks after the last IA) and changed back to 6 mg at week 20. This intermittent dose escalation potentially influenced the course and outcome of SCORAD and EASI scores measured at week 21 in favor of IA efficacy, as the worsening of skin scores at that point might have been more pronounced than without the dose modification.

In summary, these preliminary results suggest efficacy, sustainability and safety of selective extracorporeal IgE depletion using single use adsorbers in AD. Considering the small number of probands in this study which were allowed to continue their prior immunosuppressive medication, larger clinical trials are mandatory to corroborate our results, to better stratify responsive subpopulations and, in light of the promising therapeutic perspectives, to design combinatory immunomodulatory treatment plans addressing both, efficacious initial clearance, and subsequent maintenance.

## CONFLICT OF INTEREST

The authors declare that they have no conflicts of interest.

## AUTHOR CONTRIBUTIONS

D.M., C.G., M.L., J.W.B., A.K. contributed to the conception and design of study. D.M., C.G., M.L., and E.M. contributed to the data acquisition and W.H. analyzed the data. D.M. and M.L. wrote the article with the help of C.G. and J.W.B.
